# Test-retest reliability of peak location in the sensorimotor network of resting state fMRI for potential rTMS targets

**DOI:** 10.3389/fninf.2022.882126

**Published:** 2022-10-03

**Authors:** Yun-Song Hu, Juan Yue, Qiu Ge, Zi-Jian Feng, Jue Wang, Yu-Feng Zang

**Affiliations:** ^1^Center for Cognition and Brain Disorders, The Affiliated Hospital Hangzhou Normal University, Hangzhou, China; ^2^Zhejiang Key Laboratory for Research in Assessment of Cognitive Impairments, Hangzhou, China; ^3^Institutes of Psychological Sciences, Hangzhou Normal University, Hangzhou, China; ^4^Institute of Sports Medicine and Health, Chengdu Sport University, Chengdu, China

**Keywords:** inter-scanner reliability, intra-scanner reliability, ICA, SMN, peak location

## Abstract

Most stroke repetitive transcranial magnetic stimulation (rTMS) studies have used hand motor hotspots as rTMS stimulation targets; in addition, recent studies demonstrated that functional magnetic resonance imaging (fMRI) task activation could be used to determine suitable targets due to its ability to reveal individualized precise and stronger functional connectivity with motor-related brain regions. However, rTMS is unlikely to elicit motor evoked potentials in the affected hemisphere, nor would activity be detected when stroke patients with severe hemiplegia perform an fMRI motor task using the affected limbs. The current study proposed that the peak voxel in the resting-state fMRI (RS-fMRI) motor network determined by independent component analysis (ICA) could be a potential stimulation target. Twenty-one healthy young subjects underwent RS-fMRI at three visits (V1 and V2 on a GE MR750 scanner and V3 on a Siemens Prisma) under eyes-open (EO) and eyes-closed (EC) conditions. Single-subject ICA with different total number of components (20, 30, and 40) were evaluated, and then the locations of peak voxels on the left and right sides of the sensorimotor network (SMN) were identified. While most ICA RS-fMRI studies have been carried out on the group level, that is, Group-ICA, the current study performed individual ICA because only the individual analysis could guide the individual target of rTMS. The intra- (test-retest) and inter-scanner reliabilities of the peak location were calculated. The use of 40 components resulted in the highest test-retest reliability of the peak location in both the left and right SMN compared with that determined when 20 and 30 components were used for both EC and EO conditions. ICA with 40 components might be another way to define a potential target in the SMN for poststroke rTMS treatment.

## Introduction

Repetitive transcranial magnetic stimulation (rTMS), as a non-invasive neuromodulation technique, has been widely used to promote functional recovery in stroke patients (Barros Galvao et al., 2014; Aşkin et al., 2017; Dionisio et al., 2018; Bonin Pinto et al., 2019). Most stroke rTMS studies have used hand motor hotspots as stimulation targets (Sasaki et al., 2014; Diekhoff-Krebs et al., 2017; Long et al., 2018). Recent studies have demonstrated that functional magnetic resonance imaging (fMRI) task activation could also be considered a target due to its individualized precise and stronger functional connectivity (FC) with motor-related brain regions (Hulst et al., 2017). However, in stroke patients with severe hemiplegia, rTMS is unlikely to elicit motor evoked potentials in the affected hemisphere, nor is activity expected to be detected when these individuals perform an fMRI motor task using the affected limbs (Sasaki et al., 2014; Diekhoff-Krebs et al., 2017; Long et al., 2018).

Resting-state fMRI (RS-fMRI) FC is an approach to assessing the relationships among brain regions. This connectivity represents the temporal synchronization of spontaneous brain activity among distributed brain regions associated with particular functions (Fox et al., 2005; Cole et al., 2010). Over recent years, the combination of high spatial resolution functional magnetic resonance imaging (fMRI) and navigation systems allows researchers to locate the stimulation target with millimeter accuracy. Considering the variability of the individual brain, a precisely localized stimulation target is the key issue to improve the efficacy of stimulation treatment. A study defined the peak voxel of RS-fMRI seed-based FC in the superficial brain area as an rTMS target and then successfully altered FC while improving participants' memory performance (Wang et al., 2014b). Another recent study found that seed-based FC strength could predict local activity changes in the deep brain region (Feng et al., 2021). Stroke RS-fMRI studies have used seed-based FC analysis to investigate the abnormal FC of patients with damaged motor function (Park et al., 2011; Zheng et al., 2016). However, how an effective stimulation target for rTMS treatment should be defined remains unknown.

As a data-driven method, independent component analysis (ICA) decomposes whole-brain fMRI data into a given number of spatially independent components (McKeown et al., 1998; Pamilo et al., 2012). ICA has been applied in many studies, resulting in the elucidation of a host of brains intrinsic connectivity networks, such as the default mode network, visual network, sensorimotor network (SMN), language network, and auditory network (Stevens et al., 2009; Allen et al., 2011; Shirer et al., 2012; Iraji et al., 2019). RS-fMRI studies using ICA found a decreased SMN in stroke patients (Zhang et al., 2017; Zhao et al., 2018b). One of the hypotheses of the outcome of TMS in stroke treatment is to modulate sensorimotor network functions (Grefkes and Fink, 2012). Therefore, we proposed that the location of the peak voxel might be a potential stimulation target for rTMS treatment for patients with poststroke hemiplegia.

If the peak voxel is indeed a suitable stimulation target, then the reliability of the location of the peak voxel in the SMN is critical. One study reported that rTMS induced an increase in motor-cortical excitability in only 52% of healthy subjects, while the other half responded with a decrease in excitability (Hamada et al., 2013). In addition to stimulation parameters, the bias of the target may be one of the reasons for the unclear effect of rTMS. Quite a few studies have reported moderate to high test-retest reliability of ICA metrics, including well-reproducible spatial patterns (Franco et al., 2009; Meindl et al., 2010) and voxel-wise intraclass correlation (ICC) (Zuo et al., 2010). However, the reliability of the location of the peak voxel in the ICA network has not been reported. Considering the potential importance of individualized precise rTMS treatment, the reliability of peak location of single-subject ICA-derived SMN must first be assessed.

In addition, it should be noted that several factors may affect the reliability of ICC approaches. First, there is little consensus on how many components to extract for single-subject ICA. Second, RS-fMRI is unconstrained by the task and it is affected by the status of the subjects. For example, Yan and colleagues found that the FC was significantly different among the EC and EO conditions in SMN (Yan et al., 2009). Finally, the inter-scanner differences could introduce significant variability in the RS-fMRI signal (Zhao et al., 2018a). The current study investigated both intra- (test-retest) and inter-scanner reliability of the SMN peak voxel location under EO and EC conditions to evaluate whether the peak location of SMN could be a potential target for rTMS treatment.

## Materials and methods

The RS-fMRI data in the current study were acquired in previous test-retest reliability studies (Yuan et al., 2018; Zhao et al., 2018a). The current study focused on the test-retest reliability of the peak location of ICA-driven SMN, and the content of the current study had no relation to their studies.

**Figure 1:**
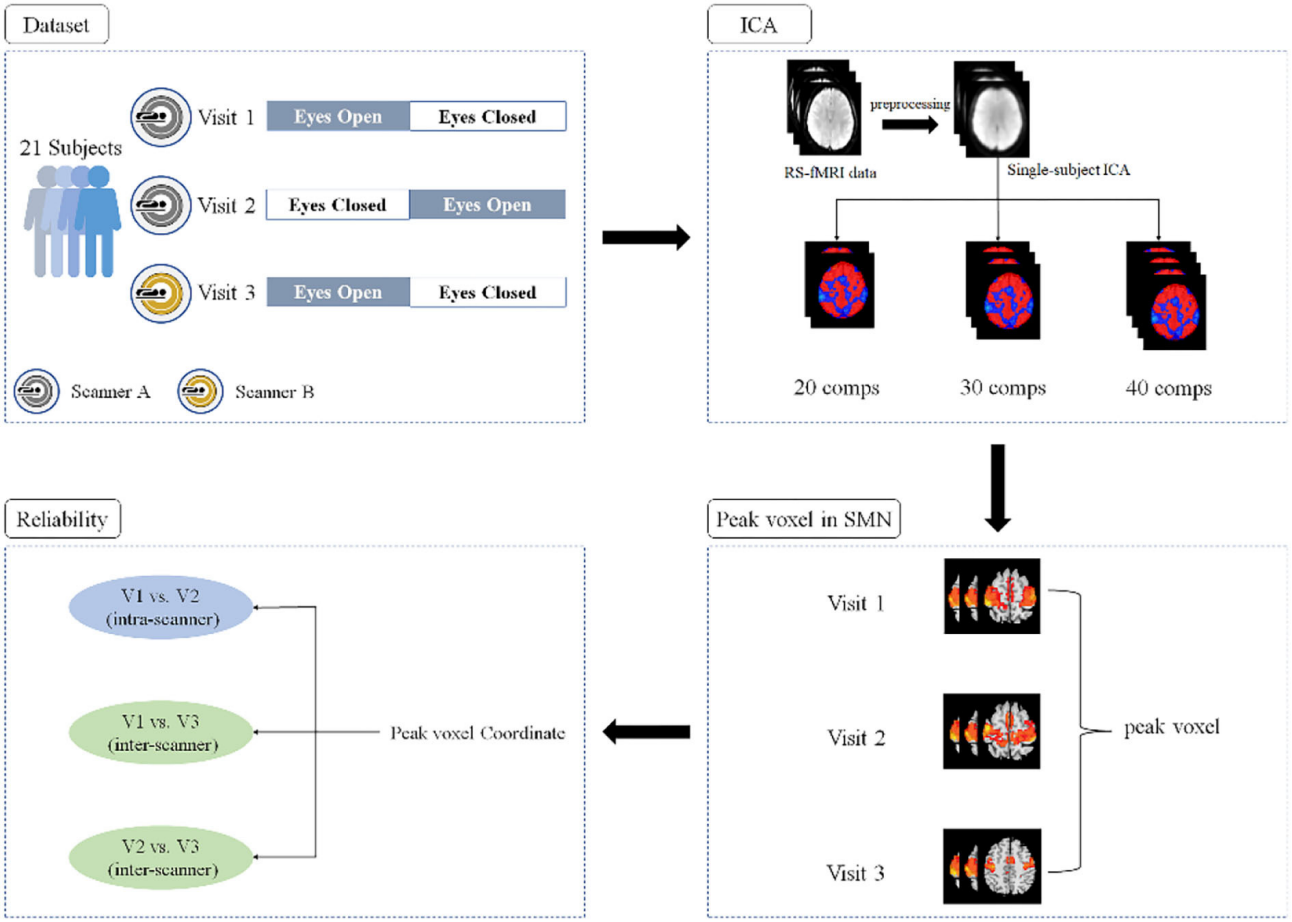
A flowchart of the experiment design and data analyses.

A flowchart of the current study is shown in Figure 1. Twenty-one healthy participants (21.8 ± 1.8 years old, 11 females) with no history of neurological or psychiatric disorders were recruited. All subjects underwent RS-fMRI scans three times; the first two were performed during the first two visits (V1 and V2, approximately 2 weeks apart) with one GE 3T scanner (MR750, GE Medical Systems, Milwaukee, WI) and the last scan was performed during the third visit (V3, approximately 8 months after V2) with a Siemens 3T scanner (Prisma, Siemens Healthineers Erlangen, Germany). For scans on both GE and Siemens scanners, a gradient-echo echo-planar imaging (EPI) pulse sequence was used for blood oxygen level-dependent (BOLD) images with the following parameters: repetition time (TR) = 2,000 ms; echo time (TE) = 30 ms; flip angle (FA) = 90°; 43 slices with interleaved acquisition; matrix = 64 × 64; field of view (FOV) = 220 mm; and acquisition voxel size = 3.44 × 3.44 × 3.20 mm. For each visit, all the participants underwent two 8-min RS-fMRI sessions (i.e., EC and EO), during which they were asked to relax and either keep their eyes open naturally (without fixation) or keep their eyes closed, not to think of anything in particular, and not to fall asleep. To minimize head movement, straps and foam pads were used to fix the participants' heads comfortably during scanning.

### Data preprocessing

Analysis of RS-fMRI data was performed by using DPABI_5.1 (C. G. Yan et al., 2016). Data preprocessing included (1) removing the first 10 volumes; (2) slice timing correction; (3) head motion correction; (4) spatial normalization to standard Montreal Neurological Institute (MNI) space by using an EPI template; and (5) spatial smoothing with a Gaussian kernel of 6-mm full width at half-maxima (FWHM).

### Single-subject level ICA

Single-subject level spatial ICA was used to separate the BOLD signal into spatially independent components using the Group ICA of fMRI Toolbox (GIFT) (http://icatb.sourceforge.net/) (Calhoun et al., 2001). The RS-fMRI studies of ICA-derived SMN usually used 20 to 40 components in recent years (Smith et al., 2009; Zuo et al., 2010; Besseling et al., 2013; Shumskaya et al., 2017; Ye et al., 2017; Thomas et al., 2019; Caspers et al., 2021; Legget et al., 2021). The current study, therefore, performed single-subject ICA with three different numbers of total components (i.e., 20, 30, and 40). Independent components were estimated by the Infomax algorithm, which is based on the principle of maximum information transfer (Bell and Sejnowski, 1995). Component stability was determined by running the ICASSO toolbox, which was used to perform 20 iterations of the ICA with randomly initialized decomposition matrices and the same convergence threshold (Himberg and Hyvarinen, 2003; Himberg et al., 2004). Then, the components of a single subject were obtained and the spatial maps were then converted into z-scores (threshold = 1).

### Identification of SMN components

SMN components were identified by using Dice coefficient quantification with an SMN mask and careful visual inspection, as described in previous studies (Akin et al., 2017; Venkataraman et al., 2021). The SMN mask includes the bilateral pre/postcentral gyri (Pre/PostCG) and supplementary motor area (SMA), which was constructed using the Harvard-Oxford probabilistic atlas from the fMRIB Software Library (FSL) (Smith et al., 2004; Makris et al., 2006). The SMN mask was transferred to the MNI space as same as fMRI data. A previous study reported that the SMN could be represented as two separate components (i.e., left and right) when evaluated by ICA (Abou-Elseoud et al., 2010). Thus, the components with the largest and second-largest Dice coefficients with the SMN mask were selected and further confirmed by visual inspection.

### ICC

The coordinates of the peak voxel within both the left and right SMN were obtained. The intra-scanner (i.e., V1 vs. V2) and inter-scanner (i.e., V1 vs. V3 and V2 vs. V3) reliability of the left and right SMN peak locations under both EC and EO conditions were estimated using ICCs according to the following equation (Shrout and Fleiss, 1979):


$$\text{ICC}=\frac{\text{MSb}\;-\;\text{MSw}}{\text{MSb}\;+\;(\text{K}\;-\;1)\;\text{MSw}}$$

where *Msb* represents the between-subject mean square of coordinate, *MSw* represents the within-subject mean square of coordinate, and *K* is the number of sessions.

The ICCs of the X, Y, and Z axes were calculated separately, and the averaged ICCs of the three axes were obtained. Although ICCs theory is never negative, ICCs estimates can take negative values when *MSb* is smaller than *MSw*. In the current study, negative values were calculated as 0 with an understanding that the true ICCs were very low in these conditions as the previous study mentioned (Ma and MacDonald, 2021).

## Results

### Single-subject SMN

**Figure 2:**
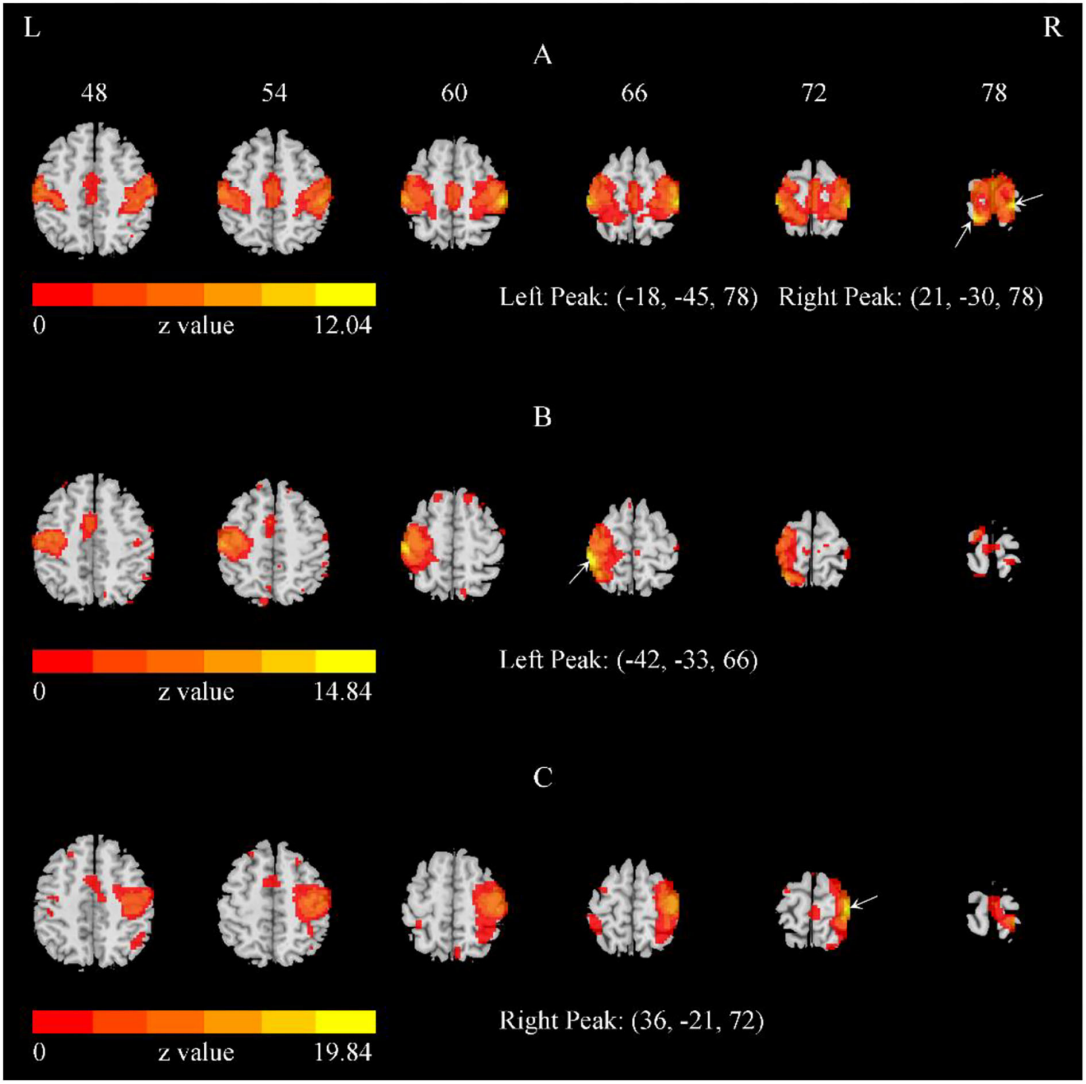
The representative SMN with the total number of components = 40. **(A)** The bilateral SMN of a representative subject with the total number of components = 40. **(B,C)** The separate left and right SMN of another subject with the total number of components = 40. The arrows indicate the peaks. Of the 378 iterations, most indicated bilateral SMN, while 23 iterations yielded the left and right SMN separately.

**Figure 3:**
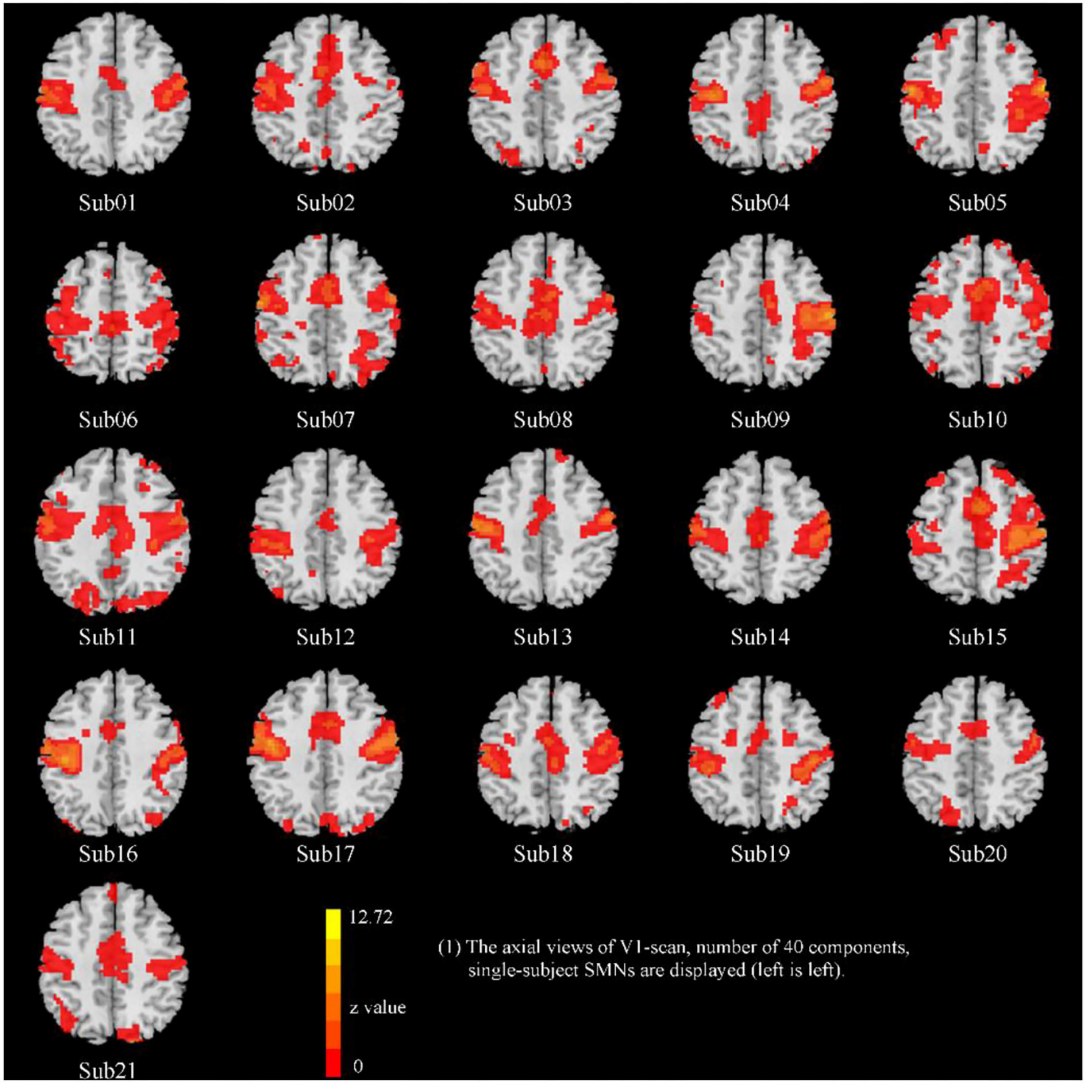
The representative SMN of 21 subjects (visit 1, total number of 40 components, EO condition).

We performed single-subject ICA 378 times: 21 (subjects) × 2 (EO and EC) × 3 (V1, V2, V3) × 3 (20, 30, and 40 total number of components). For the 378 iterations, most of the outcomes indicated the bilateral SMN (Figures 2, 3), while 23 times yielded the left and right SMN separately (The dice coefficients of the two components were close, and the spatial components of motor areas were symmetrical) (Figure 2). For the left or right component, we used the left or right side of the SMN mask to obtain the peak location.

### Intra- and inter-scanner reliability of the peak location

**Figure 4:**
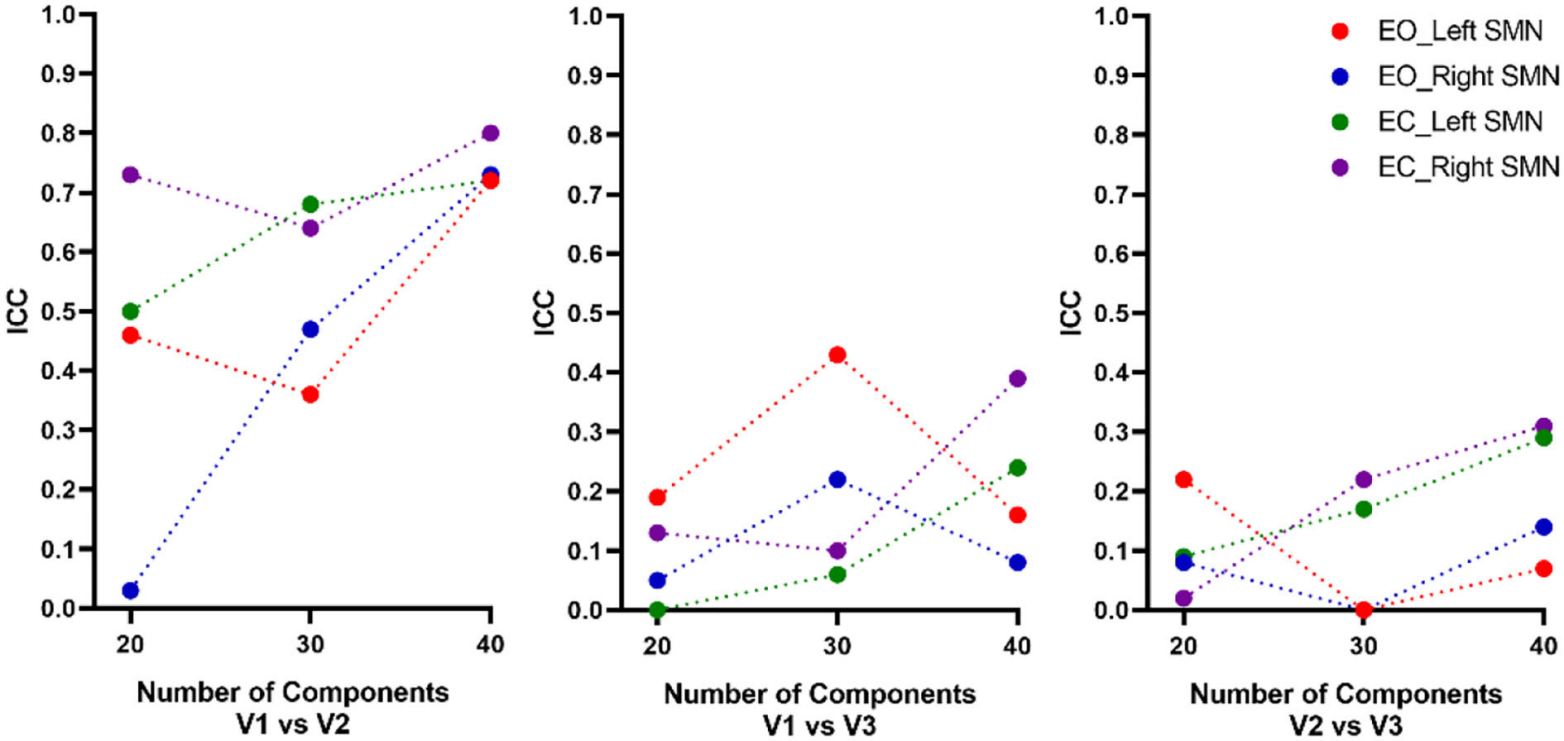
Intra- and inter-scanner test-retest reliability of the peak location of the SMN. Overall, for both EO and EC conditions, the peak location based on a total of 40 components had the highest intra-scanner (test-retest) reliability (averaged ICC = 0.72~0.8), being similar for EO and EC conditions. The 20 and 30 components had low to moderate intra-scanner reliability (averaged ICC = 0.03~0.73). The inter-scanner reliability of the total number of components was low. ICC, intraclass correlation; EO, eyes open; EC, eyes closed; V, visit.

Overall, for both EO and EC conditions, the peak location based on 40 components had the highest intra-scanner (test-retest) reliability (averaged ICC = 0.72~0.8), and was similar for EO and EC conditions (Table 1, Figure 4). The peak locations based on 20 and 30 components had low to moderate intra-scanner reliability (averaged ICC = 0.03~0.73). The intra-scanner reliability was higher than the inter-scanner reliability for the locations determined based on all the component totals under both EO and EC conditions.

**Table 1:** The intra- and inter-scanner reliability of peak voxels within the left and right SMN of EO/EC conditions.

**EC/EO** **left/right**	**Intra- and** **inter-scanner**	**Number of components**	**X**	**ICC** **Y**	**Z**	**Average**
EO left SMN	V1 vs. V2	20	0.08	0.72	0.59	0.46
		30	0.27	0.31	0.51	0.36
		40	0.69	0.59	0.87	0.72
	V1 vs. V3	20	< 0	0.57	< 0	0.19
		30	0.67	0.24	0.37	0.43
		40	0.05	0.43	< 0	0.16
	V2 vs. V3	20	< 0	0.67	< 0	0.22
		30	< 0	< 0	< 0	0.00
		40	< 0	0.21	< 0	0.07
EO right SMN	V1 vs. V2	20	< 0	0.08	< 0	0.03
		30	0.47	0.41	0.52	0.47
		40	0.62	0.81	0.76	0.73
	V1 vs. V3	20	0.13	0.01	< 0	0.05
		30	0.59	< 0	0.08	0.22
		40	< 0	0.23	< 0	0.08
	V2 vs. V3	20	< 0	< 0	0.23	0.08
		30	< 0	< 0	< 0	0.00
		40	< 0	0.41	< 0	0.14
EC left SMN	V1 vs. V2	20	0.34	0.65	0.51	0.50
		30	0.71	0.57	0.76	0.68
		40	0.77	0.64	0.75	0.72
	V1 vs. V3	20	< 0	< 0	< 0	0.00
		30	< 0	0.18	< 0	0.06
		40	0.19	0.26	0.26	0.24
	V2 vs. V3	20	0.27	< 0	< 0	0.09
		30	< 0	0.51	< 0	0.17
		40	< 0	0.65	0.23	0.29
EC right SMN	V1 vs. V2	20	0.77	0.69	0.73	0.73
		30	0.69	0.72	0.52	0.64
		40	0.83	0.76	0.80	0.80
	V1 vs. V3	20	0.39	< 0	< 0	0.13
		30	< 0	0.31	< 0	0.10
		40	0.19	0.57	0.41	0.39
	V2 vs. V3	20	0.06	< 0	< 0	0.02
		30	< 0	0.65	< 0	0.22
		40	0.49	0.40	0.04	0.31

The ICCs of the X, Y, and Z axes were calculated separately, and negative values for the average were calculated as 0. ICC, intraclass correlation; EO, eyes open; EC, eyes closed; V, visit.

We also calculated the Euclidean distance of the peak location between V1 and V2 (i.e., intra-scanner distance). Tables 2–4 showed the Euclidean distance of each subject based on 20, 30, and 40 components, the peak voxels with lower ICC (20 and 30 number of components) showed very large Euclidean distance (18.85−47 mm and 21.63–33.5 mm), while the peak voxels with highest ICC (40 number of components) showed smaller Euclidean distance (18.80−21.45 mm). It should be noted that only 41% of peaks had an intra-scanner distance of 0–10 mm. This means that although the ICC was higher, the peak distance between the two visits was large.

**Table 2:** The Euclidean distance (mm) between the peak locations of V1 and V2 within the left/right SMN of EO/EC conditions (20 components).

**Subject ID**	**EO_left SMN**	**EO_right SMN**	**EC_left SMN**	**EC_right SMN**
Sub001	4	9	45	3
Sub002	31	45	14	0
Sub003	30	24	3	5
Sub004	63	59	86	3
Sub005	10	23	52	0
Sub006	3	7	24	35
Sub007	12	85	4	25
Sub008	16	58	28	60
Sub009	52	59	26	25
Sub011	38	45	69	4
Sub012	18	4	0	11
Sub013	7	68	30	3
Sub014	87	52	38	7
Sub015	0	74	0	0
Sub016	59	0	59	54
Sub017	57	81	3	22
Sub018	5	0	22	72
Sub019	38	77	23	0
Sub020	26	103	3	28
Sub021	71	67	22	20
Mean	31.35	47	27.55	18.85
STD	25.86	31.68	24.39	21.73

Sub10 was excluded because the SMN component could not be identified.

**Table 3:** The Euclidean distance (mm) between the peak locations of V1 and V2 within the left/right SMN of EO/EC conditions (30 components).

**Subject ID**	**EO_left SMN**	**EO_right SMN**	**EC_left SMN**	**EC_right SMN**
Sub001	4	16	45	0
Sub002	42	54	0	0
Sub003	0	22	3	7
Sub004	28	3	25	4
Sub005	45	46	52	26
Sub006	77	31	24	3
Sub007	5	69	42	10
Sub008	7	0	64	77
Sub009	33	14	3	31
Sub011	0	23	-	-
Sub012	14	7	4	11
Sub013	38	43	30	7
Sub014	47	42	54	35
Sub015	55	21	28	0
Sub016	22	3	23	85
Sub017	59	64	22	18
Sub018	5	0	34	57
Sub019	35	20	21	0
Sub020	83	82	5	0
Sub021	71	61	54	40
Mean	33.5	31.05	28.05	21.63
STD	26.41	25.13	19.75	26.50

Sub10 was excluded because the SMN component could not be identified.

**Table 4:** The Euclidean distance between the peak locations of V1 and V2 within the left/right SMN of EO/EC conditions (40 components).

**Subject ID**	**EO_left SMN**	**EO_right SMN**	**EC_left SMN**	**EC_right SMN**
Sub001	4	16	3	7
Sub002	27	0	41	0
Sub003	0	12	3	12
Sub004	14	0	4	24
Sub005	52	0	3	0
Sub006	32	23	17	13
Sub007	7	79	35	31
Sub008	3	24	4	55
Sub009	20	0	39	3
Sub011	58	33	42	47
Sub012	0	7	78	4
Sub013	19	27	36	51
Sub014	62	25	4	16
Sub015	30	21	7	26
Sub016	0	62	18	0
Sub017	19	4	4	40
Sub018	31	15	21	18
Sub019	24	19	7	26
Sub020	24	0	21	3
Sub021	3	25	5	0
Mean	21.45	19.60	19.60	18.80
STD	19.02	20.61	20.00	18.11

Sub10 was excluded because the SMN component could not be identified.

## Discussion

To the best of our knowledge, the current RS-fMRI study was the first to evaluate the test-retest reliability of the peak voxel location using ICA; these outcomes have potential clinical significance for precise targeting of rTMS. We found that the use of 40 total components had the highest intra-scanner (test-retest) reliability for the peak voxel location of the SMN compared to that obtained when using 20 and 30 components.

### Peak voxel location in the SMN as the rTMS target

The current study proposed that the location of the voxel with peak FC might be a potential stimulation target for rTMS treatment of poststroke hemiplegia. Motor dysfunction in stroke patients was viewed as a system-level disruption of brain networks (Wang et al., 2010, 2014a) without a common local lesion. Previous RS-fMRI studies have found decreased brain activity of the SMN in stroke patients by using either ICA (Zhang et al., 2017; Zhao et al., 2018b) or seed-based FC analysis (Zheng et al., 2016; Tsuchimoto et al., 2019). Another study conducted an assessment to determine the peak voxel in the ipsilesional precentral gyrus during group-level task activation that could be used as a seed region of interest (ROI) for RS-fMRI FC analysis and found decreased connectivity of the ipsilesional M1 with the contralesional SMN (Zheng et al., 2016). Seed-based FC has been successfully used to define the rTMS target for modulating brain functions in healthy subjects (Wang et al., 2014b). Although abnormal FC of the SMN was reported in the above-mentioned studies, it is certainly not easy to define a common seed ROI for FC-guided rTMS of poststroke hemiplegia due to its diverse lesion foci. Precentral activation-based FC is unable to comprehensively and objectively reflect all poststroke motor functions, especially the functions of the SMN. Moreover, there is compelling evidence that the neural correlates driving hand movements are rather dependent on the regulation of the interactions among regions of the motor network (Solodkin et al., 2004; Swinnen and Wenderoth, 2004; Wiesendanger and Serrien, 2004). Importantly, the precentral gyrus may no longer be a representation of the SMN when hemiplegia occurs after a stroke. Graph theory studies have reported that the degree and betweenness centrality of some hub nodes significantly decreased when the function of the network was affected; hence, these nodes were no longer hubs (Pandit et al., 2013; Aerts et al., 2016). Although the reliability of the SMN peak location in the current study was based on healthy subjects and not on stroke patients, the previous study had indicated that the response to plasticity-inducing intervention might depend on how the stimulation interacts with the pre-existing connectivity in the functional network (Diekhoff-Krebs et al., 2017). And for stroke patients, the peak location of the ipsilateral hemisphere SMN is still the node with the strongest functional connectivity. Thus, defining a location that represents the neural activity of the whole SMN can yet be regarded as a kind of method to identify rTMS therapeutic targets in structurally damaged brains.

As a data-driven method, ICA could provide complementary and valuable information for investigating the mechanism of impaired function of the brain network in diverse neurologic diseases. For example, the most consistent finding obtained by using seed-based FC in previous RS-fMRI studies was that patients with motor impairment after stroke show decreased connectivity of the ipsilesional precentral gyrus with the contralesional SMN (Park et al., 2011; Zheng et al., 2016), while ICA studies reported decreased connectivity in both the ipsilesional and contralesional precentral and postcentral gyrus (S1) (Zhang et al., 2017; Zhao et al., 2018b). As the core network is vulnerable to stroke lesions, ICA-derived SMN may yield results that lead to a more comprehensive understanding of network impairment in stroke patients with hemiplegia compared to the results obtained from seed-based FC. Moreover, we found that 51% of the total 740 peaks (348 for both left and right, and 16 peaks were excluded because of an unidentified SMN component) were in the precentral gyrus, 46% of the peaks were in the postcentral gyrus, and 3% of the peaks were in the SMA. Notably, studies of stroke have demonstrated that abnormal local brain activity within the postcentral gyrus was associated with somatosensory deficits (Liang et al., 2018; Jiang et al., 2019), while increased fMRI task activation was found in the precentral gyrus and appeared in the postcentral gyrus along with motor function recovery (Luft et al., 2004). In addition, the peak location of the ICA network represents the position with the strongest temporal synchronization of spontaneous brain activity with other regions within the functional network (Damoiseaux et al., 2008), and the peak location of the postcentral as the strongest functional connectivity within SMN may be the hub that modulates the network, not just the precentral gyrus. The findings mentioned earlier lead to one hypothesis regarding whether the SMN peak in the precentral or postcentral gyrus could be a potential target for rTMS to modulate SMN functions and further improve motor symptoms.

### Test-retest reliability of the peak location in the SMN

The current study proposed that the peak location in the ICA-derived SMN could be a potential stimulation target for rTMS treatment of poststroke hemiplegia. However, the peak location may vary due to the number of components, and therefore, the current study tested the intra- and inter-scanner reliability of the peak SMN location in which the total number of components was a factor. Previous studies focused on RS-fMRI ICA found that functionally connected regions would be split into separate subnetworks with a high number of components (Van de Ven et al., 2004), while the separated subnetworks would be mixed with various components, such as noise, when too few components were set (Van de Ven et al., 2004; Bartels and Zeki, 2005). To date, there is no accepted number of components for the separation of individual SMNs, and previous studies have used different numbers of components for different purposes. The current study measured the test-retest reliability of individualized SMNs with three different numbers of total components that were commonly used in previous ICA studies. Among the three different numbers of total components, we found that the use of 40 components resulted in the highest intra-scanner reliability of the SMN peak location. Given the number of components has been an uncertain question of ICA, it could be a reference to obtaining a stable location as an rTMS target. Further analysis of Euclidean distance between two visits showed that the peak voxels with lower ICC (20 and 30 number of components) shower very large Euclidean distance, while the peak voxels with highest ICC showed smaller Euclidean distance. Although several subjects showed large distance (>10 mm), the average Euclidean distance of 40 components is much smaller than 20 and 30. Future studies could focus on improving the reliability of single-subject ICA network peak locations. It should be noted that the reliability of the SMN peak location in the current study was based on healthy subjects and not on stroke patients. To verify the feasibility of the proposed method on stroke patients, future studies should measure the test-retest reliability of peak voxels on stroke patients. The high test-retest reliability is a suitable characteristic that makes this location a potential rTMS target for hemiplegia poststroke and other neuropsychiatric disorders.

## Limitations

The first limitation of the current study was the relatively short RS-fMRI scan (8 min). It has been reported that the reliability of FC could be greatly improved by increasing the scanning time (Mueller et al., 2015). Longer scanning times in future studies may improve the reliability of the peak location of the ICA-driven network.

The second limitation was that the current results showed only 41% peaks of number of 40 components had an intra-scanner Euclidean distance of 0–10 mm. Although the ICC was higher, according to the results of Euclidean distance analysis, some subjects' peak distance between two visits was large. The specific reasons for this difference are not clear for now, and future studies could focus on improving the reliability of single-subject ICA network peak locations.

The third limitation was that the inter-scanner spans short and long term but does not balance the order of scanning machines. Future studies should balance the scanning order to explore inter-scanner reliability.

## Data availability statement

The datasets presented in this study can be found in online repositories. The names of the repository/repositories and accession number(s) can be found at: https://www.nitrc.org/.

## Ethics statement

The studies involving human participants were reviewed and approved by Ethics Committee of the Center for Cognition and Brain Disorders (CCBD) at Hangzhou Normal University (HZNU). The patients/participants provided their written informed consent to participate in this study.

## Author contributions

Y-SH, JW, and Y-FZ analyzed the data and wrote the paper. JY, QG, and Z-JF processed the data. All authors contributed to the article and approved the submitted version.

## Funding

This study was supported by NSFC (82071537, 81520108016), Key Realm R&D Program of Guangdong Province (2019B030335001), Key Medical Discipline of Hangzhou, and The Cultivation Project of the Province-Leveled Preponderant Characteristic Discipline of Hangzhou Normal University (18JYXK046, 20JYXK004).

## Conflict of interest

The authors declare that the research was conducted in the absence of any commercial or financial relationships that could be construed as a potential conflict of interest.

## Publisher's note

All claims expressed in this article are solely those of the authors and do not necessarily represent those of their affiliated organizations, or those of the publisher, the editors and the reviewers. Any product that may be evaluated in this article, or claim that may be made by its manufacturer, is not guaranteed or endorsed by the publisher.
